# Enhancing CAR-T Efficacy in Large B-Cell Lymphoma with Radiation Bridging Therapy: A Real-World Single-Center Experience

**DOI:** 10.3390/curroncol32030173

**Published:** 2025-03-17

**Authors:** Eva Laverdure, Luigina Mollica, Imran Ahmad, Sandra Cohen, Silvy Lachance, Olivier Veilleux, Maryse Bernard, Eve-Lyne Marchand, Jean-Sébastien Delisle, Lea Bernard, Mélissa Boileau, Tony Petrella, Sarah-Jeanne Pilon, Philippe Bouchard, Denis-Claude Roy, Lambert Busque, Isabelle Fleury

**Affiliations:** 1 Department of Medicine, Institut Universitaire d’Hémato-Oncologie et de Thérapie Cellulaire, Hôpital Maisonneuve-Rosemont, CIUSSS de l’Est-de-l’Île-de-Montréal, l, Montréal, QC HIT 2M4, Canada; eva.laverdure@usherbrooke.ca (E.L.); luigina.mollica.med@ssss.gouv.qc.ca (L.M.); imran.ahmad.1@umontreal.ca (I.A.); sandra.cohen.med@ssss.gouv.qc.ca (S.C.); silvy.lachance@umontreal.ca (S.L.); olivier.veilleux.med@ssss.gouv.qc.ca (O.V.); js.delisle@umontreal.ca (J.-S.D.); denis-claude.roy.med@ssss.gouv.qc.ca (D.-C.R.);; 2 Department of Hemato-Oncology, Hôpital Fleurimont, Centre Hospitalier Universitaire de Sherbrooke, CIUSSS de l’Estrie, Faculté de Médecine et des Sciences de la Santé, Université de Sherbrooke, Sherbrooke, QC J1H 5N4, Canada; 3 Faculty of Medicine, Université de Montréal, Montréal, QC H2V 0B3, Canada; maryse.bernard.med@ssss.gouv.qc.ca (M.B.); emarchand.hmr@ssss.gouv.qc.ca (E.-L.M.); tony.petrella.med@ssss.gouv.qc.ca (T.P.); sarah-jeanne.pilon.med@ssss.gouv.qc.ca (S.-J.P.); 4 Department of Radiation Therapy, Institut Universitaire d’Hémato-Oncologie et de Thérapie Cellulaire, Hôpital Maisonneuve-Rosemont, CIUSSS de l’Est-de-l’Île-de-Montréal, Montréal, QC H1T 2M4, Canada; 5 Department of Pathology, Institut Universitaire d’Hémato-Oncologie et de Thérapie Cellulaire, Hôpital Maisonneuve-Rosemont, CIUSSS de l’Est-de-l’Île-de-Montréal, Montréal, QC H1T 2M4, Canada; 6 Department of Pharmacy, Institut Universitaire d’Hémato-Oncologie et de Thérapie Cellulaire, Hôpital Maisonneuve-Rosemont, CIUSSS de l’Est-de-l’Île-de-Montréal, Montréal, QC H1T 2M4, Canada

**Keywords:** CAR-T, large B-cell lymphoma, bridging therapy, radiation therapy

## Abstract

One challenge of chimeric antigen receptor T-cell therapy (CAR-T) for relapsed or refractory large B-cell lymphoma (LBCL) is achieving disease control during manufacturing. We report real-word outcomes of 100 patients treated with axicabtagene ciloleucel (axi-cel, *n* = 50) or tisagenlecleucel (tisa-cel, *n* = 50) at our center. Most patients received bridging therapy (BT) with 48 undergoing radiation BT (RBT) and 32 receiving systemic BT (SBT). The best overall response rate (ORR) was 84% (78% complete response (CR)) for axi-cel and 60% (42% CR) for tisa-cel. At a median follow-up of 16 months, 12-month progression-free survival (PFS) and overall survival (OS) were 72% and 82% for axi-cel, compared to 35% and 57% for tisa-cel. By the bridging approach, 12-month PFS was 60% with RBT, 59% without BT and 35% with SBT (*p* = 0.06). Notably, axi-cel patients without lymphoma progression during manufacturing (*n* = 24) achieved 12-month PFS and OS rates of 91% and 96%, respectively. Axi-cel was associated with more cytokine release syndrome (92% vs. 66%, *p* = 0.003) and neurotoxicity (all-grade 56% vs. 10%, *p* < 0.001, grade ≥ 328% vs. 4%, *p* = 0.002). Multivariate analysis identified RBT as independently associated with improved PFS (HR 0.46, 95% CI 0.22–0.96). Pending prospective validation, RBT shows promise for improving CAR-T outcomes in LBCL.

## 1. Introduction

CD19-targeted chimeric antigen receptor T-cell (CAR-T) therapy has transformed the treatment landscape for relapsed or refractory (R/R) large B-cell lymphoma (LBCL) in patients who have failed conventional chemotherapy. Axicabtagene ciloleucel (axi-cel) and tisagenlecleucel (tisa-cel) are both approved for R/R LBCL after at least two prior lines of therapy, based on the phase 2 trials ZUMA-1 and JULIET, which demonstrated durable disease control in 30–40% of patients [1,2]. Despite less stringent eligibility criteria, real-world data have globally reproduced the outcomes observed in these pivotal studies [3,4,5,6,7,8].

CAR-T is also now approved as second-line therapy for LBCL with primary refractory disease or relapse within 12 months, demonstrating its superiority over standard salvage chemotherapy followed by autologous stem cell transplantation [9,10]. Ongoing clinical trials are evaluating the role of axi-cel in frontline settings, aiming to improve outcomes for high-risk patients who may benefit from earlier intervention with CAR-T cell therapy [11].

However, rapidly progressive disease remains a major barrier to successful CAR-T therapy that can preclude infusion following apheresis [12]. Many patients require bridging therapy (BT) to maintain disease control during the vein-to-vein time from apheresis to CAR-T infusion. Selecting an optimal bridging strategy remains a significant challenge [13,14,15], as evidence on its impact on CAR-T outcomes is limited and conflicting, leaving clinicians without clear guidance [12,16,17].

In this study, we report real-world data from the first 100 patients treated with commercial axi-cel and tisa-cel at our institution, and present data suggesting a favorable impact of prioritizing radiation therapy as a bridging strategy.

## 2. Materials and Methods

### 2.1. Study Design and Patient Selection

We retrospectively analyzed the first 100 patients with R/R LBCL treated with commercial CAR-T at Hôpital Maisonneuve-Rosemont, an academic center affiliated to Université de Montreal, Canada. Clinical data were collected through a comprehensive electronic chart review. Patient eligibility for the pivotal trials was retrospectively evaluated.

Axi-cel and tisa-cel are the two anti-CD19 CAR-T approved and reimbursed by public health insurance in Canada. As per coverage regulation, adults with diffuse large B-cell lymphoma (DLBCL), high-grade B-cell lymphoma (HGBCL), transformed follicular lymphoma (tFL) or primary mediastinal large B-cell lymphoma (PMBCL) R/R after two or more lines of systemic therapy were eligible.

BT, defined as lymphoma-directed therapy administered after apheresis and before lymphodepletion chemotherapy, included systemic BT (SBT) and radiation BT (RBT). Comprehensive RBT targeted all disease sites identified on the positron emission tomography–computed tomography (PET-CT) before apheresis, whereas partial RBT targeted only selected sites of disease such as symptomatic, bulky or at risk of anatomical compression.

Patients were restaged with PET-CT before lymphodepletion. The required washout period before CAR-T infusion was 14 days for chemotherapy and radiation therapy and five days for steroids.

### 2.2. Assessments and Endpoints

Disease response was evaluated locally using PET-CT according to Lugano criteria [18], and assessments scheduled at 1 and 3 months post-infusion and subsequently as needed until complete response (CR) or confirmed progressive disease (PD). Efficacy and safety cohorts included all 100 infused patients. Cytokine release syndrome (CRS) and immune effector cell-associated neurotoxicity syndrome (ICANS) were graded according to the American Society for Transplantation and Cellular Therapy (ASTCT) criteria [19].

Survival endpoints were measured from the date of CAR-T infusion until the date of the first event, with censoring for patients without event at last contact. Progression-free survival (PFS) was defined as the time to lymphoma relapse, progression or death from any cause. Overall survival (OS) was defined as the time to death from any cause. Non-relapse mortality (NRM) was defined as death without relapse or progression.

### 2.3. Statistical Analysis

Comparisons between cohorts were performed using Kruskal–Wallis and Fisher’s exact tests. Probabilities of PFS and OS were estimated using the Kaplan–Meier method with log-rank tests. Cumulative incidences of progression and NRM were estimated using the cumulative incidence function with competing risks and compared using Gray’s test.

Predictors of efficacy and safety were tested using the Cox proportional hazards regression model for PFS and OS and the Fine-Gray subdistribution hazard model for NRM. Dichotomous outcomes were modeled using logistic regression. Multivariate analysis (MVA) incorporated significant variables from univariate analysis, considering collinearity and clinical relevance. Confidence intervals are reported at 95%, and reported *p* values were two-sided, with a *p* value of <0.05 considered statistically significant. Statistical analyses were performed using R 4.1.0 (R Core Team, 2021).

More details are available in the Online Supplementary Materials.

## 3. Results

### 3.1. Patient Characteristics

Tisa-cel was first approved in Canada, with the first infusion on 14 August 2019, followed by axi-cel, with the first infusion on 4 December 2019. From August 2019 to March 2023, 106 patients underwent apheresis, of whom 100 (94%) were infused. The six patients not receiving CAR-T had experienced prohibitive disease progression despite BT (3 SBT and 3 RBT) and died from lymphoma progression at a median of 57 days (range, 14–74 days) post-apheresis. There was no manufacturing failure.

Fifty patients were treated with axi-cel and fifty with tisa-cel. The median age at infusion was 59 years (range, 20–81 years). Most patients had DLBCL (59%), followed by tFL (22%), HGBCL (12%) and PMBCL (7%). *MYC* and *BCL2* and/or *BCL6* rearrangements were present in 18% of cases. Two patients had secondary central nervous system involvement. Seventy percent of patients were primary refractory (defined as failure to achieve CR or relapse within six months of first-line therapy). Sixteen percent had received prior high-dose therapy followed by autologous stem cell transplant (HDT-ASCT). The median number of prior lines of systemic therapy was two (range, 2–5) with CAR-T administered as third-line therapy in 75%, fourth line in 19% and fifth or sixth line in 6%. Four patients had received prior CD3xCD20 bispecific antibody, all within a year of CAR-T infusion.

At apheresis, 66% of patients had advanced-stage disease, 18% had bulky disease (≥7.5 cm tumor mass) and 35% had a revised International Prognostic Index (R-IPI) of 3–5.

### 3.2. Patient Characteristics by CAR-T Product

Patients treated with tisa-cel were older (median 64 vs. 56 years; *p* = 0.001) and had a longer vein-to-vein time (median 49 days (range, 24–127) vs. 36 (range, 24–126); *p* < 0.001) than patients treated with axi-cel. Among axi-cel recipients, 86% would have been ineligible for the ZUMA-1 trial (including 40% for reason(s) other than the use of BT) compared to 28% of tisa-cel recipients who would have been ineligible for the JULIET trial (Supplemental Tables S1 and S2). All PMBCL were treated with axi-cel per reimbursement criteria. Other baseline characteristics, including BT modality, were similar between the two CAR-T groups (Supplemental Table S3). Key comorbidities are described in Supplemental Table S4. CAR-T selection evolved toward increased use of axi-cel during the study period (Supplemental Figure S1).

### 3.3. Bridging Therapy

BT was administered to 80% of patients (Table 1 and Supplemental Figure S2): 48 received RBT and 32 received SBT. Five patients required two lines of BT due to rapid disease progression and two because of prolonged COVID-19 infection delaying infusion.

At apheresis, SBT patients had more advanced-stage disease (*p* = 0.002), elevated LDH (*p* = 0.001) and poor risk R-IPI of 3–5 (*p* = 0.002) compared to RBT or no BT patients. All patients with bulky disease at apheresis received BT (9 RBT and 9 SBT).

Median vein-to-vein time was 44 days for RBT (range, 24–92), 43 days for SBT (range, 25–127) and 37 days for no BT (range, 26–106).

Among the RBT cohort (*n* = 48), 75 sites were irradiated: abdomen/pelvis (*n* = 32), thorax (*n* = 22), head/neck (*n* = 7), axilla (*n* = 6), extremity/soft tissue (*n* = 5) and bone (*n* = 3). Twenty-eight patients were irradiated at a single site (nineteen of them were comprehensive), fourteen at two sites, five at three sites and one at four sites. The median radiation dose delivered to each site was 25 Gy (range, 4–30 Gy) with a median fraction size of 5 Gy (range, 2–8 Gy). The most common regimens were 25 Gy in five fractions (*n* = 43 sites) and 20 Gy in five fractions (*n* = 13 sites). No patient had previously received lymphoma-directed radiation therapy. Dexamethasone was administered concurrently to 29 patients. All planned radiation doses were delivered and no delays in CAR-T infusion were attributed to radiation-induced adverse events.

Compared to patients who received partial RBT (*n* = 18), comprehensive RBT patients (*n* = 30) had less advanced-stage disease at apheresis (33% vs. 89%; *p* < 0.001) and at infusion (23% vs. 89%; *p* < 0.001).

In the SBT cohort (*n* = 32), 84% received conventional chemotherapy. The most used regimens were ICE (*n* = 17) and IVAC (*n* = 4), with rituximab incorporated in three patients. One patient received bendamustine/polatuzumab vedotin. Four patients received steroids alone.

### 3.4. Response to BT Prior to CAR-T Infusion

The early effect of BT was evaluated with PET-CT before lymphodepletion. Lymphoma progression rates were comparable across bridging modalities, with 46% of patients infused in PD after RBT and 59% infused in PD after SBT. Radiation therapy was able to achieve good local control with only four in-field progressions during the bridging period across all 75 irradiated sites. Two patients required a second line of BT due to RBT out-of-field disease progression during the manufacturing period.

Within the RBT cohort, patients who received comprehensive RBT were less likely to be in PD at CAR-T infusion compared to those who received partial RBT (33 vs. 67%; *p* = 0.037).

### 3.5. CAR-T Efficacy

The best overall response rate (ORR) was 72% with a 60% CR rate (CRR) (Table 2). Axi-cel achieved superior results (ORR 84%, CRR 78%) compared to tisa-cel (ORR 60%, CRR 42%). The median time to CR was 2.8 months (95% CI 1.3–9.6).

Fifteen of the twenty-seven patients (56%) with initial PR and one of the two patients (50%) with stable disease (SD) at one month converted to CR at a median of 100 days after infusion (range, 56–650 days). Four patients experienced late CR (>6 months after infusion), equally distributed across CAR-T products: three patients with RBT achieved CR at 8, 9 and 21 months and one with no BT achieved CR at 7 months.

**Figure 1:**
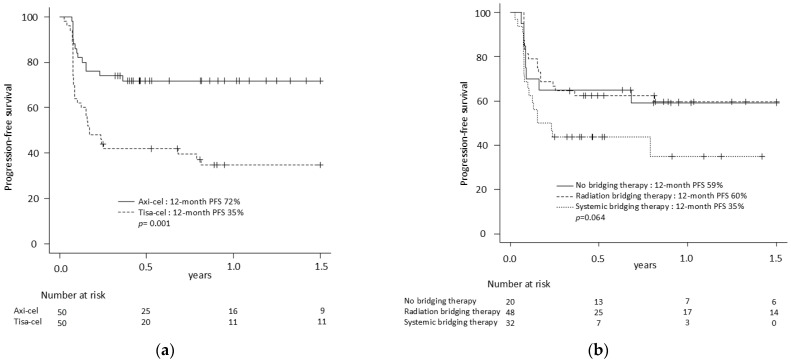
Progression-free survival according (**a**) to CAR-T (**b**) to bridging therapy. Abbreviations: PFS: progression-free survival; CAR-T: chimeric antigen receptor T-cell therapy; axi-cel: axicabtagene ciloleucel; tisa-cel: tisagenlecleucel.

The median follow-up was 16.0 months (95% CI 11.4–21.2). The median duration of CR was not reached (NR), with only five relapses observed among CR patients: three after tisa-cel (89, 195 and 240 days after achieving CR) and two after axi-cel (27 and 105 days after achieving CR). The median PFS was NR with a 12-month estimate of 53%. Patients treated with axi-cel had significantly longer PFS compared to tisa-cel (median NR vs. 2.0 months; 12-month PFS 72% vs. 35%; *p* = 0.001) (Figure 1a).

The median OS was 26.0 months with a 12-month OS estimated of 69%. There was a trend toward an OS benefit with axi-cel compared to tisa-cel (median OS NR vs. 22.2 months, 12-month 82% vs. 57%, *p* = 0.083; Supplemental Figure S3).

### 3.6. Outcomes Based on BT, RBT Field and Disease Status at Infusion

Despite having unfavorable disease characteristics, patients who received BT had similar survival outcomes to those who did not, with a 12-month PFS of 59% for BT versus 51% for no BT (*p* = 0.557), while 12-month OS was 74% versus 68% (*p* = 0.274), respectively (Supplemental Figure S4). Patients who received RBT or no BT had numerically higher PFS and statistically higher OS compared to those who received SBT, with a 12-month PFS of 60% vs. 59% vs. 35% (*p* = 0.064) and a 12-month OS 79% vs. 74% vs. 52% (*p* = 0.001) (Figure 1b, Supplemental Figure S5). Compared to partial RBT, comprehensive RBT led to higher CR rates post-CAR-T infusion (77% vs. 50%; *p* = 0.039) with a trend toward higher PFS (12-month 70% vs. 44%, *p* = 0.095) and similar OS (12-month 85% vs. 70%, *p* = 0.232) (Supplemental Figure S6).

Of the eleven patients infused in CR (per PET-CT before lymphodepletion), one of the three treated with tisa-cel and all eight treated with axi-cel remained in CR at a median follow-up of 169 days (range, 122–298). CAR-T expansion was not monitored, but eight patients experienced CRS, with one grade 3 event, and seven experienced ICANS, with three grade 3 and one grade 4 events.

**Figure 2:**
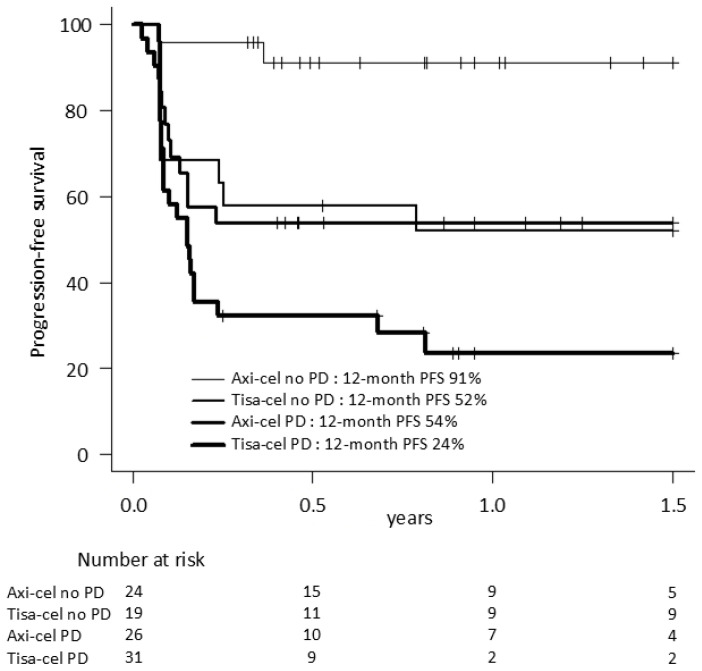
Progression-free survival according to CAR-T and disease status at infusion. PFS: progression-free survival; CAR-T: chimeric antigen receptor T-cell therapy; axi-cel: axicabtagene ciloleucel; tisa-cel: tisagenlecleucel, PD: progressive disease prior lymphodepletion.

Patients without lymphoma progression between apheresis and infusion (in CR, PR or SD on PET-CT prior lymphodepletion) (no PD) had improved outcomes compared to those infused in PD. Axi-cel patients with no PD at infusion had the best outcomes with a 12-month PFS of 91% vs. 54% if infused in PD (*p* = 0.003). For tisa-cel, this difference did not reach statistical difference (12-month PFS 52% with no PD vs. 24% with PD, *p* = 0.067) (Figure 2; OS data in Supplemental Figure S7).

Following CAR-T failure, 71% of patients (29/41) received salvaged therapy, amounting to 66 subsequent lines of treatment (median of 1 per patient; range, 1–6). Salvage therapies included clinical trials (24%), radiation therapy (32%), chemotherapy-based (14%), polatuzumab vedotin-based (12%) and tafasitamab-based (2%). Among these, 13 patients received lenalidomide-based therapy, 5 received CD3xCD20 bispecific antibodies and 2 received checkpoint inhibitors through clinical trials or special access programs. Three patients underwent allogeneic stem cell transplant as a second, fourth and fifth line of salvage, and all progressed within 100 days post-transplant.

### 3.7. Safety and Toxicity

The rates of grades 1–3 and grade 3 CRS were 79% and 6%, respectively, while grades 1–4 and grades 3–4 ICANS occurred in 33% and 16%, respectively (Table 3). No grades 4–5 CRS or grade 5 ICANS were observed. Rates of CRS (all grade) and ICANS (all grade and grades 3–4) were higher with axi-cel compared to tisa-cel. All-grade ICANS was more frequent with SBT than with RBT or no BT (*p* = 0.008). Management of CRS and/or ICANS included tocilizumab in 51% and corticosteroids in 33% of the entire cohort. Seven patients (one tisa-cel and six axi-cel) received anakinra for steroid-refractory ICANS.

The median duration of hospitalization post-infusion was 15 days (range, 7–70) after axi-cel and 11 days (range, 0–38) after tisa-cel. Among the seventeen patients receiving outpatient tisa-cel, six (35%) required hospitalization. The median length of hospital stay after tisa-cel infusion was 13 days (range, 7–38) if infused as inpatients and 11 days (range, 5–14) if infused as outpatients.

Within 30 days of infusion, intensive care unit (ICU) admission was required in 26% of patients, with a median ICU stay of 4 days (range, 1–28). ICU admission rates were 32% for axi-cel and 20% for tisa-cel (*p* = 0.254) and were more frequent after SBT and RBT compared to no BT (38% vs. 27% vs. 5%, respectively, *p* = 0.033).

By 3 months after infusion, NRM occurred in four patients (4%), equally distributed between CAR-T products, and were all due to infections within 30 days post-infusion: *Pneumocystis jirovecii* pneumonia (PJP), nosocomial pneumonia, invasive *Candida tropicalis* and neutropenic enterocolitis. All cases occurred in patients who had received SBT. Only one late NRM occurred and was secondary to COVID-19 pneumonia 249 days after infusion.

Axi-cel was associated with more severe neutropenia at 1 and 3 months and more severe thrombocytopenia at 1 month. SBT was associated with more severe cytopenias across all lineages at 1 month and more severe thrombocytopenia at 3 months. During the first 3 months following infusion, 33 patients required red blood cell transfusion (median, two per patient; range, 1–13) and 23 received platelet transfusions (median, six per patient; range, 1–23). Three months after infusion, 17% of evaluable patients still required growth factor support. Replacement therapy for hypogammaglobulinemia was administered to 8% of patients for severe or repeated infections.

In the first 3 months after infusion, 32 clinically significant infections occurred in 28 patients: 22 bacterial infections, 4 fungal infections (1 invasive candidemia, 1 invasive aspergillosis, 1 proven and 1 suspected PJP) and 6 viral infections (4 COVID-19, 1 cytomegalovirus and 1 respiratory syncytial virus pneumonia). Bacterial infections were more frequent after SBT (28% vs. 17% for RBT vs. 5% for no BT), while fungal infections were exclusively observed in patients receiving SBT. During follow-up, twenty patients developed COVID-19, including four recurrent infections, four hospitalizations and one fatal outcome.

### 3.8. Predictors of Efficacy and Safety

In MVA (Table 4), inferior PFS was associated with CAR-T product (HR 4.10 for tisa-cel (vs. axi-cel), 95% CI 1.95–8.62), elevated LDH at apheresis (HR 5.69, 95% CI 1.72–18.82) and PD between apheresis and infusion (HR 2.13, 95% CI 1.04–4.35). RBT was associated with a reduced risk of progression (HR 0.46, 95% CI 0.22–0.96). Despite collinearity between RBT and advanced-stage disease, MVA indicated an overriding benefit of RBT. In the same model, elevated LDH at apheresis was the only factor significantly associated with inferior OS (HR 6.17, 95% CI 1.40–27.18). Comprehensive RBT (vs partial RBT) and vein-to-vein time did not significantly impact PFS or OS.

Grade ≥ 3 CRS was associated with bulky disease at infusion (HR 7.55 95% CI 1.35–42.10) and grade ≥ 3 ICANS was less frequent with tisa-cel (HR 0.11 95% CI 0.02–0.50). Other factors, including age > 65, PD between apheresis and infusion, high LDH, advanced-stage disease, ferritin > 650 ug/L, CRP > 30 mg/L, platelet count < 175 × 10^9^/L and bridging modality, were not significantly associated with grade ≥ 3 CRS or ICANS.

## 4. Discussion

CAR-T has established itself as a new standard of care for patients with R/R LBCL. Our analysis of the first 100 patients treated with commercially available CAR-T in third -line or later reveals significantly higher rates of ORR, CRR and PFS with axi-cel compared to tisa-cel. Notably, the CRR of 78% for axi-cel and 12-month PFS of 72% compares favorably to the pivotal trials ZUMA-1 [20] (CRR 58%, 12-month PFS 44%) and JULIET [21] (CRR 40%, 12-month PFS 35%) as well as to other real-world studies [3,4,5,6,7,8]. Importantly, patients in our study displayed unfavorable features including 70% primary refractory disease and 57% that would have been ineligible to their respective registration trial.

Manufacturing time remains a major challenge with CAR-T [22]. High tumor burden and poor performance status at infusion are associated with inferior outcomes in both clinical trials and real-world settings [23]. Successful cytoreduction through BT may circumvent some of these limitations and may represent a marker of a more favorable biology [24]. However, BT efficacy in R/R LBCL is unpredictable and may introduce additional toxicities.

Among our cohort, 80% of patients received BT, with 46% and 59% demonstrating disease progression before infusion despite RBT and SBT, respectively. These findings underscore the limitations of BT in these high-risk patients.

During the study period, only six patients who underwent apheresis failed to reach infusion. This low attrition rate may reflect strict performance status (ECOG of 0 to 1) mandated for CAR-T eligibility as well as tailored BT strategies determined through multidisciplinary boards.

Disease status at CAR-T infusion significantly impacted outcomes with a 30–40% decrease in 12-month PFS with both CAR-T products for patients with PD between apheresis and infusion. By contrast, axi-cel patients without progression during the manufacturing period achieved an unparallelled 12-month PFS of 91%. Consistent with other studies [25,26], elevated LDH at apheresis, a surrogate marker for high tumor burden, was a strong independent predictor of inferior PFS and OS.

Radiation therapy offers the advantage of local tumor control in chemorefractory LBCL with limited toxicity. Beyond its cytoreductive effect, RT exerts pleiotropic biological effects on the tumor and its microenvironment, potentially enhancing the anti-tumor effect of CAR-T [27]. RT has emerged as a promising BT based on retrospective studies, but the evidence is limited by heterogeneity in patient selection, field sizes, doses and fractionation [28,29,30,31].

In our cohort of chemorefractory patients in third line or more, with no access to novel immunotherapies such as polatuzumab-vedotin [13], RBT was generally our preferred bridging modality to maintain disease control during CAR-T manufacturing. RBT provided excellent field control for the 48 patients with disease amenable to this approach.

Most patients received hypofractionated regimens with a median dose of 25 Gy, a lower dose than usually required for curative ablation in aggressive lymphoma. This regimen of 25 Gy/5 fractions was informed by clinical experience [32] and recommendations from the International Lymphoma Radiation Oncology Group (ILROG) proposed for consolidative RT during the COVID-19 pandemic [33]. In our cohort, RBT offered interesting features in terms of safety with no toxicity postponing planned infusion and no increased rate of severe CRS, ICANS and NRM.

Comprehensive RBT, delivered to 30 patients, showed superior outcomes (CRR 77%, 12-month PFS 70%) compared to partial RBT (CRR 50%, 12-month PFS 44%), although not statistically significant, consistent with trends reported in previous studies. Pinnix et al. and Hubbeling et al. also identified a trend toward improved PFS with comprehensive RBT in a cohort of 17 and 25 patients exposed to RBT [12,17]. Manzar et al. reported a superior PFS and OS in a univariate analysis for the 22 patients who received comprehensive RBT compared to the 18 who received partial RBT [34]. Ladbury et al. also demonstrated a benefit to comprehensive RBT in eight patients who achieved a 1-year PFS and OS of 100% compared to the 1-year PFS of 9% and OS of 46% in the eleven patients who had focal RBT [16]. Furthermore, an ILROG multi-institutional study of 115 patients reported an improved PFS in 40 patients who received comprehensive RBT in MVA [28]. Furthermore, Saifi et al. reported improved local control and event-free survival with comprehensive RBT compared to no BT in patients with fewer than five involved disease sites before CAR-T, indicating a potential added benefit of RBT even when accounting for disease burden [35].

At the time of our analysis, CAR-T therapy was only available in the third line or later. With CAR-T now accessible in the second line, our findings remain relevant, especially for patients with limited disease amenable to comprehensive RBT.

Our retrospective review highlights the challenges in analyzing the role of bridging therapy in real-world settings. Direct comparison between comprehensive and partial RBT groups is limited by inherent selection bias introduced by differences in disease burden between these groups. The absence of data on metabolic tumor volume (MTV) on PET/CT is another limitation, and future studies should incorporate MTV assessment to better evaluate the effect of BT on outcomes. It is likely that the choice of bridging therapy and response to it will differ in a less heavily pretreated second-line population, not yet refractory to platinum-based salvage, or in the context of access to novel immunotherapy. Data from larger prospective studies are required to validate our findings and to identify clinical predictors that can determine which patients will benefit most from RBT [36].

Despite the limitations associated with a single-center retrospective analysis and selection bias related to both the choice of BT and CAR-T product; our results add to the growing evidence supporting the synergistic effect of RT as a bridging modality with granular data. RBT may be especially advantageous for patients with refractory lymphoma, disease amenable to comprehensive RBTand/or limited access to novel therapies [5,13].

## 5. Conclusions

Our single-institution cohort of 100 patients with R/R LBCL treated with axi-cel and tisa-cel supports the real-world benefit of CAR-T with high remission rate and acceptable toxicity. The results provide compelling evidence supporting RBT as a safe and efficacious bridging strategy that can mitigate the adverse prognostic implications of requiring BT for CAR-T in the third-line setting. Prospective trials are needed to refine the optimal RT dosing, fractionation, field sizes and timing relative to CAR-T infusion to further optimize patient outcomes through better cytoreduction and/or immune modulation.

## Figures and Tables

**Table 1:** Patients’ characteristics for all cohort and according to bridging therapy.

	All Patients *N =* 100	No BT *N =* 20	RBT *N =* 48	SBT *N =* 32	*p*-Value *
Age, median (range)	59 (20–81)	61 (20–78)	59 (30–81)	62 (21–78)	0.62
CAR-T, *n* (%)					0.23
Axi-cel	50 (50)	9 (45)	21 (44)	20 (63)	
Tisa-cel	50 (50)	11 (55)	27 (56)	12 (38)	
Histology, *n* (%)					0.05
DLBCL	59 (59)	12 (60)	23 (48)	24 (75)	
tFL	22 (22)	5 (25)	11 (23)	6 (19)	
PMBCL	7 (7)	2 (10)	3 (6)	2 (6)	
HGBCL	12 (12)	1 (5)	11 (23)	0 (0)	
Primary refractory, *n* (%)	70 (70)	12 (60)	36 (75)	22 (69)	0.46
Ineligibility to pivotal trial, *n* (%)	57 (57)	4 (20)	27 (56)	26 (81)	<0.01
At apheresis, *n* (%)					
Stage III or IV	66 (66)	11 (55)	26 (54)	29 (91)	<0.01
Bulky disease	18 (18)	0 (0)	9 (19)	9 (28)	0.04
Elevated LDH	62 (62)	7 (35)	28 (58)	27 (84)	<0.01
≥2 extranodal sites	23 (23)	3 (15)	7 (15)	13 (41)	0.06
At lymphodepletion, *n* (%)					
Stage III or IV	61 (61)	14 (70)	23 (48)	24 (75)	0.03
Bulky disease	14 (14)	1 (5)	7 (15)	6 (19)	0.38
Elevated LDH	55 (55)	9 (45)	23 (48)	23 (72)	0.07
≥2 extranodal sites	27 (27)	2 (10)	11 (23)	14 (44)	<0.01
Status at infusion, *n* (%)					
PD	57 (57)	16 (80)	22 (46)	19 (59)	
SD	13 (13)	4 (20)	7 (15)	2 (6)	0.03
PR	19 (19)	0 (0)	13 (27)	6 (19)	
CR	11 (11)	0 (0)	6 (13)	5 (16)	

* Comparison of all 3 groups. Abbreviations: CAR-T: chimeric antigen receptor T-cell therapy; BT: bridging therapy; RBT: radiation BT; SBT: systemic BT; Axi-cel: axicabtagene ciloleucel; Tisa-cel: tisagenlecleucel; DLBCL: diffuse large B-cell lymphoma; tFL: transformed follicular lymphoma; PMBCL: primary mediastinal large B-cell lymphoma; HGBCL: high-grade B-cell lymphoma; LDH: lactate dehydrogenase; PD: progressive disease; SD: stable disease; PR: partial response; and CR: complete response.

**Table 2:** Efficacy outcomes for all cohorts and according to CAR-T and BT.

	All Patients *N =* 100	Axi-cel *N =* 50	Tisa-cel *N =* 50	No BT *N =* 20	RBT *N =* 48	SBT *N =* 32
Best response (%)						
ORR	72	84 *	60 *	70	79	63
CR	60	78 *	42 *	60	67	50
Median PFS, mo	NR	NR *	2.0 (1.0–9.7) *	NR	NR	2.3 (1.0-NE)
12-month PFS (95% CI)	53% (42–61)	72% (57–82) *	35% (22–48) *	59% (34–77)	60% (44–72)	35% (16–55)
Median OS, mo	26.0 (17.3-NR)	NR	22.2 (7.0-NE)	37.2 (8.2-NE) †	NR †	12.5 (3.4–26.0) †
12-month OS (95% CI)	69% (58–77)	82% (68–91)	57% (42–70)	74% (48–88) †	79% (63–88) †	52% (31–69) †

* *p* < 0.05 axi-cel vs. tisa-cel; † *p* < 0.05 no BT vs. RBT vs. SBT. Abbreviations: CAR-T: chimeric antigen receptor T-cell therapy; BT: bridging therapy; Axi-cel: axicabtagene ciloleucel; Tisa-cel: tisagenlecleucel; RBT: radiation BT; SBT: systemic BT; mo: months; ORR: overall response rate; CR: complete response; PFS: progression-free survival; OS: overall survival; NR: not reached; and NE: not estimable.

**Table 3:** Safety outcomes for the entire cohort and according to CAR-T and BT.

Safety Outcome	All Patients *N =* 100	Axi-cel *N =* 50	Tisa-cel *N =* 50	No BT *N =* 20	RBT *N =* 48	SBT *N =* 32
CRS, %						
Any grade	79	92 *	66 *	85	77	78
Grade ≥ 3	6	6	6	5	2	13
ICANS, %						
Any grade	33	56 *	10 *	15 †	27 †	53 †
Grade ≥ 3	16	28 *	4 *	5	15	25
ICU transfer within 30 days, %	26	32	20	5	27	38
Grade ≥ 3 at 1 month, % Neutropenia Anemia Thrombopenia	42 20 31	65 * 27 45 *	18 * 14 16 *	25 † 0 † 15 †	33 † 15 † 19 †	67 † 43 † 60 †
Grade ≥ 3 at 3 months, % Neutropenia Anemia Thrombopenia	17 4 10	29 * 7 11	3 * 0 8	29 0 0 †	12 2 7 †	18 9 23 †
NRM at 3 months	4	4	4	0 †	0 †	13 †

* *p* < 0.05 axi-cel vs. tisa-cel; † *p* < 0.05 no BT vs. RBT vs. SBT. Abbreviations: CAR-T: chimeric antigen receptor T-cell therapy; Axi-cel: axicabtagene ciloleucel; Tisa-cel: tisagenlecleucel; BT: bridging therapy; RBT: radiation BT; SBT: systemic BT; CRS: cytokine release syndrome; ICANS: immune effector cell associated neurotoxicity syndrome; ICU: intensive care unit; and NRM: non-related mortality.

**Table 4:** Multivariate analysis for PFS and OS.

Clinical Variable	Progression Free Survival			Overall Survival		
	HR	95% CI	*p*-Value	HR	95% CI	95% CI
Tisa-cel (vs. axi-cel)	4.10	1.95–8.62	<0.01	1.98	0.89–4.41	0.10
Elevated LDH at apheresis	5.69	1.72–18.82	<0.01	6.17	1.40–27.18	0.02
Stage III-IV at apheresis	1.33	0.54–3.26	0.54	2.22	0.88–5.62	0.09
RBT	0.46	0.22–0.96	0.04	0.51	0.24–1.09	0.08
PD at infusion	2.13	1.04–4.35	0.04	2.07	0.96–4.45	0.06

Abbreviations: PFS: progression-free survival; OS: overall survival; Tisa-cel: tisagenlecleucel; Axi-cel: axicabtagene ciloleucel HR: hazard ratio, LDH: lactate dehydrogenase; RBT: radiation bridging therapy; and PD: progressive disease prior lymphodepletion.

## Data Availability

The original contributions presented in this study are included in the article/Supplementary Materials. Further inquiries can be directed to the corresponding author.

## Supplementary Materials

The following supporting information can be downloaded at https://www.mdpi.com/article/10.3390/curroncol32030173/s1, Supplementary methods; Table S1: Reason(s) for ZUMA-1 ineligibility for axi-cel treated patients; Table S2: Reason(s) for JULIET ineligibility for tisa-cel treated patients; Table S3: Patients characteristics according to CAR-T; Table S4: Comorbidities according to CAR-T product; Figure S1: CAR-T product use over time; Figure S2: Bridging therapy use over time; Figure S3: Overall survival according to CAR-T; Figure S4: Progression-free survival (a) and overall survival (b) according to bridging therapy or no bridging therapy; Figure S5: Overall survival according to bridging therapy; Figure S6: Progression-free survival (a) and overall survival (b) according to the field of radiation bridging therapy; Figure S7: Overall survival according to CAR-T and lymphoma status at infusion.
