# Supplementary material for: Enhancing CAR-T Efficacy in Large B-Cell Lymphoma with Radiation Bridging Therapy: A Real-World Single-Center Experience
Source: Curr Oncol. 2025 Mar 17;32(3):173. doi: 10.3390/curroncol32030173 (PMC11941054; doi:10.3390/curroncol32030173)
Supplement: Supplementary file 1 [file curroncol-32-00173-s001.zip › curroncol-3437692-supplementary.pdf]

Supplementary methods

CAR-T reimbursement criteria:

Patients were required to have an Eastern Cooperative Oncology Group performance status (ECOG) of 0 to 1, estimated glomerular filtration rate of ≥45 ml/min/1.73m^2, left ventricular ejection fraction of ≥45%, pulse oxygenation >91% on room air, neutrophils >1x10^{9}L and platelets >50x10^9/L and serum alanine aminotransferase (ALT) ≤ 5 times the upper limit of normal (ULN)at apheresis. Patients were screened for central nervous system (CNS) lymphoma with imaging and, whenever feasible, with lumbar puncture using flow cytometry analysis.

Bridging therapy:

Bridging indication and modality were selected during a multidisciplinary tumor board by hematologists and radiation oncologists who reviewed each patient's radiological evolution throughout previous therapies. RBT feasibility was considered for every case and was generally favored for localized disease or for dominant or symptomatic lesion and/or at risk of organ compression. SBT was reserved for patients with widespread disease without a dominant critical lesion.

Multivariable analysis:

The following variables were selected for univariate analysis (UVA): age >65 years, ineligibility to pivotal trial, histology (tFL vs HGBCL vs PMBCL), primary refractory disease, prior HDT-ASCT, bendamustine exposure prior apheresis, >3 prior LOT, advanced stage at apheresis, tumor burden at apheresis and infusion (LDH, bulky disease >7,5 cm), type of CAR-T, BT (yes vs no; RBT vs SBT; RBT vs SBT/no BT; comprehensive RBT vs other BT/no BT), vein-to-vein time (<28 days vs 28-40 days vs >40 days), status at infusion as assessed by PET-CT restaging prior LD (PD vs no PD), CRP >30 mg/L at infusion, ferritin >650 ug /L at infusion, platelet <175 x10^9/L at infusion.

Supplementary Tables and Figures

Table S1: Reason(s) for ZUMA-1 ineligibility for axi-cel treated patients

Reason(s) (not mutually exclusive)	*N* (%)<br>*n*=50 axi-cel
Bridging therapy	41 (82)
Creatinine clearance <60 ml/min	4 (8)
Cardiac ejection fraction <50%	0 (0)
Baseline saturation <93% on room air	1 (2)
Serum ALT >2.5 ULN	1 (2)
Central nervous involvement	0 (0)
ANC <1000/Ul, platelet <75,000/uL	4 (8)
Clinically significant pleural or pericardiac effusion	4 (8)
History of malignancy other than nonmelanoma skin cancer or carcinoma in situ unless disease free for ≥ 3 years	0 (0)
Presence of indwelling drain	4 (8)
DVT or PE within 6 months	10 (20)
Unstable angina or myocardial infarction within 12 months	0 (0)
Autoimmune disease requiring systemic immunosuppression within 24 months	2 (4)
HIV positive	1 (2)

Abbreviations: axi-cel: axicabtagene ciloleucel; ALT: alanine aminotransferase; ULN: upper limit of normal; ANC: absolute neutrophil count; DVT: deep vein thrombosis; PE: pulmonary embolism; and HIV: human immunodeficiency virus.

Table S2: Reason(s) for JULIET ineligibility for tisa-cel treated patients

Reason(s) (not mutually exclusive)	*N* (%)<br>*n*=50 tisa-cel
Creatinine clearance <60 ml/min	7 (14)
Cardiac ejection fraction <45%	0 (0)
Baseline saturation <92% on room air	1 (2)
ALT >5 ULN	0 (0)
ANC <1000/Ul, platelet <50,000/uL	1 (2)
Central nervous involvement	2 (4)
Unstable angina or myocardial infarction within 6 months	1 (2)
History of malignancy except non-melanoma skin cancer or carcinoma in situ or completely resected and in complete remission for ≥5 years	3 (6)
T-cell/histocyte rich large B-cell lymphoma	1 (2)

Abbreviations: tisa-cel: tisagenlecleucel; ALT: alanine aminotransferase; ULN: upper limit of normal; and ANC: absolute neutrophil count.

Table S3: Patients characteristics according to CAR-T

	**Axi-cel** *n*=50	**Tisa-cel** *n*=50	***P***
Age (years), median (range)	56 (20-74)	64 (23-81)	<0.01
>65 years, n (%)	12 (24)	24 (48)	0.02
Histology, n (%)			0.04
DLBCL	27 (54)	32 (64)	
tFL	11 (22)	11 (22)	
PMBCL	7 (14)	0 (0)	
HGBCL	5 (10)	7 (14)	
Primary refractory, n (%)	37 (74)	33 (66)	0.51
≥3 prior lines, n (%)	8 (16)	17 (34)	0.06
Prior HDT-ASCT, n (%)	7 (14)	9 (18)	0.79
Prior bendamustine, n (%)	6 (12)	10 (20)	0.41
Ineligibility to the pivotal trial, n (%)	43 (86)	14 (28)	<0.01
At apheresis, n (%)			
Stage III or IV	31 (62)	35 (70)	0.53
Bulky disease	10 (20)	8 (16)	0.80
Elevated LDH	31 (62)	31 (62)	1.00
≥2 extranodal sites	8 (16)	15(30)	0.21
R-IPI 3-5	13 (26)	22 (44)	0.09
Bridging therapy	41 (82)	39 (78)	0.23
SBT	20 (40)	12 (24)	
RBT	21 (42)	27 (54)	
Comprehensive RBT	15 (30)	15 (30)	
At lymphodepletion, n (%)			
Stage III or IV	28 (56)	33 (66)	0.41
Bulky disease	7 (14)	7 (14)	1.00
Elevated LDH	30 (60)	25 (50)	0.42
≥2 extranodal sites	9 (18)	18(36)	0.14
Status at infusion, n (%)			
PD	26 (52)	31 (62)	0.42
SD	6 (12)	7 (14)	
PR	10 (20)	9 (18)	
CR	8 (16)	3 (6)	

Abbreviations: CAR-T: chimeric antigen receptor T-cell therapy; axi-cel: axicabtagene ciloleucel; tisa-cel: tisagenlecleucel; DLBCL: diffuse large B-cell lymphoma; tFL: transformed follicular lymphoma; PMBCL: primary mediastinal large B-cell lymphoma; HGBCL: high-grade B-cell lymphoma; HDT-ASCT: high-dose therapy and autologous stem cell transplant; LDH: lactate dehydrogenase; R-IPI: revised international prognostic index; SBT: systemic bridging therapy;

RBT: radiation bridging therapy; PD: progressive disease; SD: stable disease; PR: partial response;and CR: complete response.

Table S4: Comorbidities according to CAR-T product

N (%)	Axi-cel (*n*=50)	Tisa-cel (*n*=50)
Coronary artery disease	0 (0)	4 (8)
Arrythmias	3 (6)	7 (14)
Cardiac insufficiency	0 (0)	0 (0)
Hypertension	9 (18)	22 (44)
COPD/asthma	8 (16)	6 (12)
Dyslipidemia	13 (26)	17 (34)
Diabetes	3 (6)	9 (18)
Obesity (BMI ≥30)	6 (12)	8 (16)
Chronic renal insufficiency	2 (4)	2 (4)
Cerebrovascular disease	0 (0)	1 (2)
Venous thrombosis	6 (12)	6 (12)
Cirrhosis, hepatitis or portal hypertension	5 (10)	7 (14)
Psychiatric disorder	7 (14)	6 (12)

Abbreviations: CAR-T: chimeric antigen receptor T-cell therapy; axi-cel: axicabtagene ciloleucel; tisa-cel: tisagenlecleucel; COPD: chronic obstructive pulmonary disease; and BMI: body mass index.

Figure S1: CAR-T product use over time

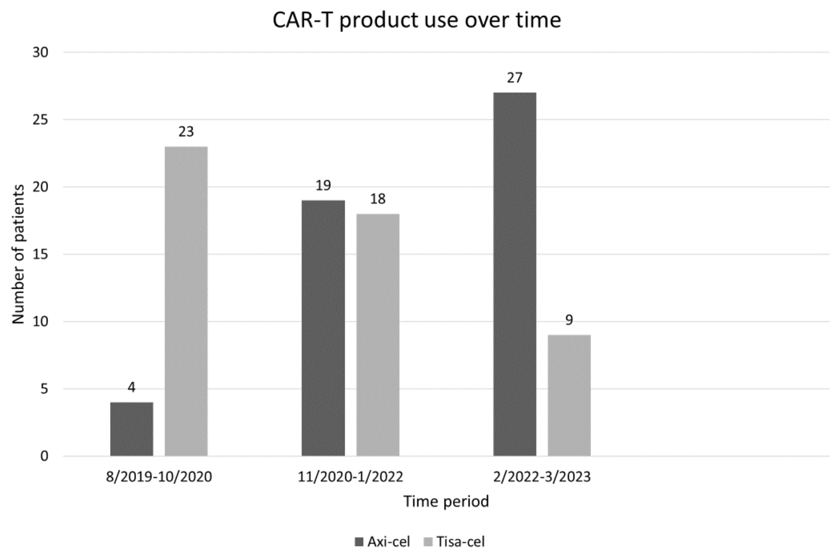

Abbreviations: CAR-T: chimeric antigen receptor T-cell therapy; axi-cel: axicabtagene ciloleucel; and tisa-cel: tisagenlecleucel.

Figure S2: Bridging therapy over time

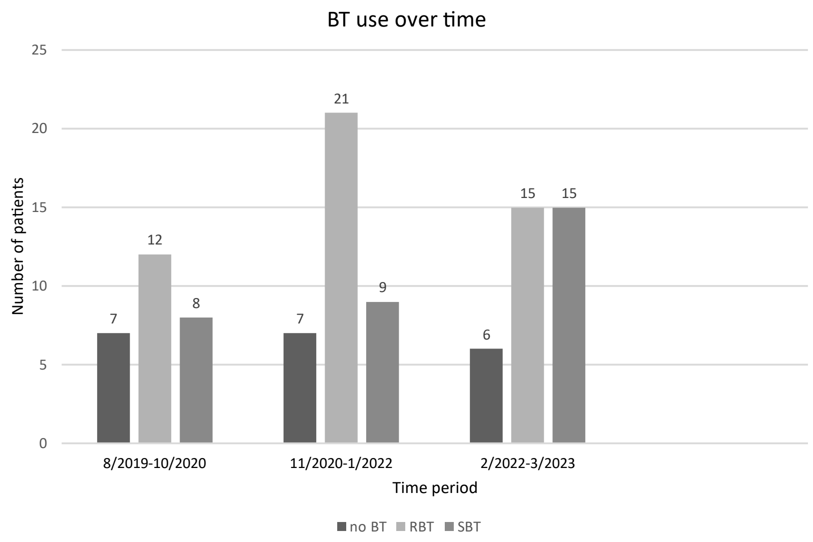

Abbreviations: BT: Bridging therapy; RBT: radiation BT; and SBT: systemic BT.

Figure S3: Overall survival according to CAR-T

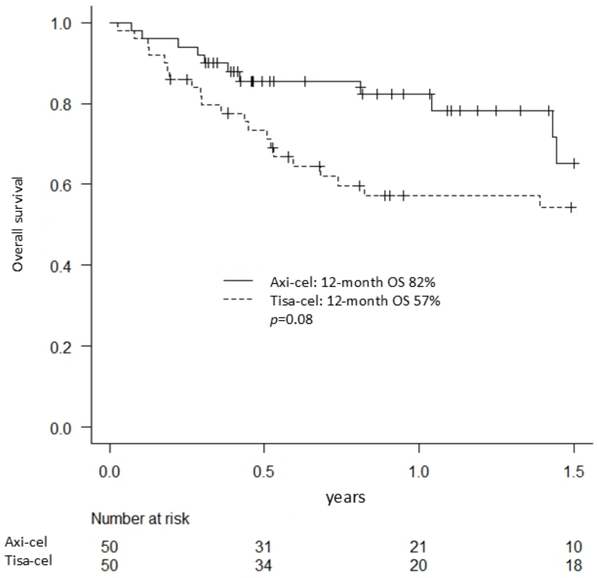

Abbreviations: OS: overall survival; CAR-T: chimeric antigen receptor T-cell therapy; axi-cel: axicabtagene ciloleucel; and tisa-cel: tisagenlecleucel.

Figure S4: Progression-free survival (a) and overall survival (b) according to bridging therapy or no bridging therapy

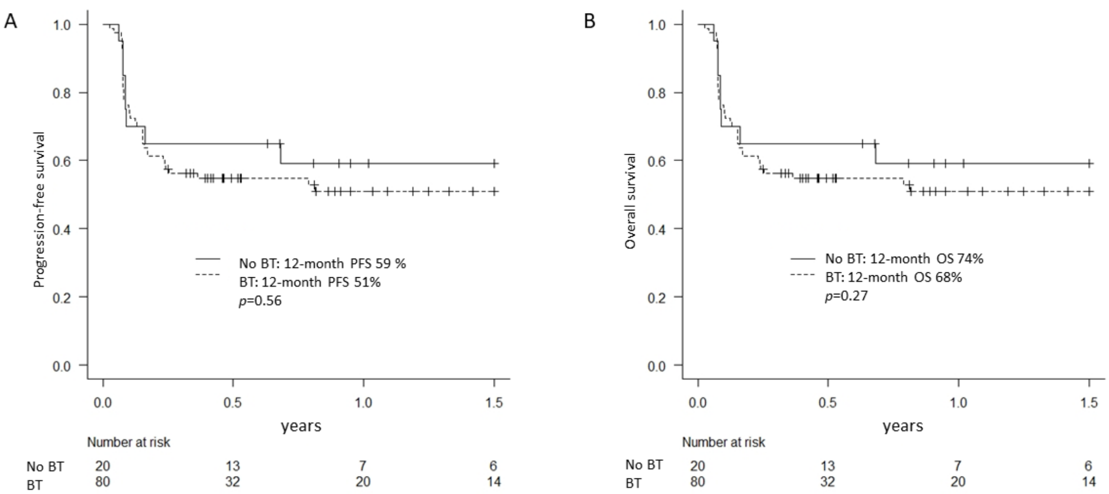

Abbreviations: PFS: progression-free survival; OS: overall survival; and BT: bridging therapy.

Figure S5: Overall survival according to bridging therapy

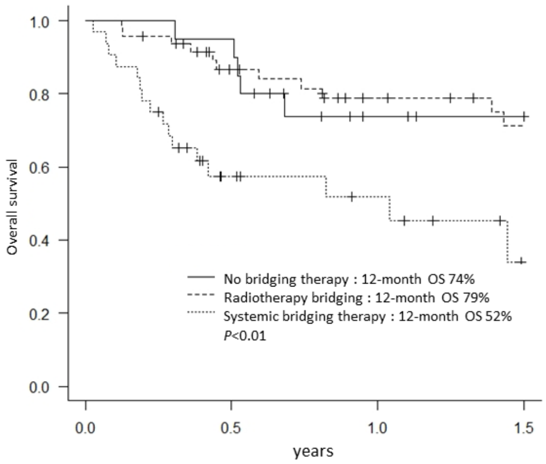

Number at risk

	0.0	0.5	1.0	1.5
No bridging therapy	20	19	9	7
Radiotherapy bridging	48	34	24	19
Systemic bridging therapy	32	12	8	2

Abbreviations: OS: overall survival.

Figure S6: Progression-free survival (a) and overall survival (b) according to the field of radiation bridging therapy

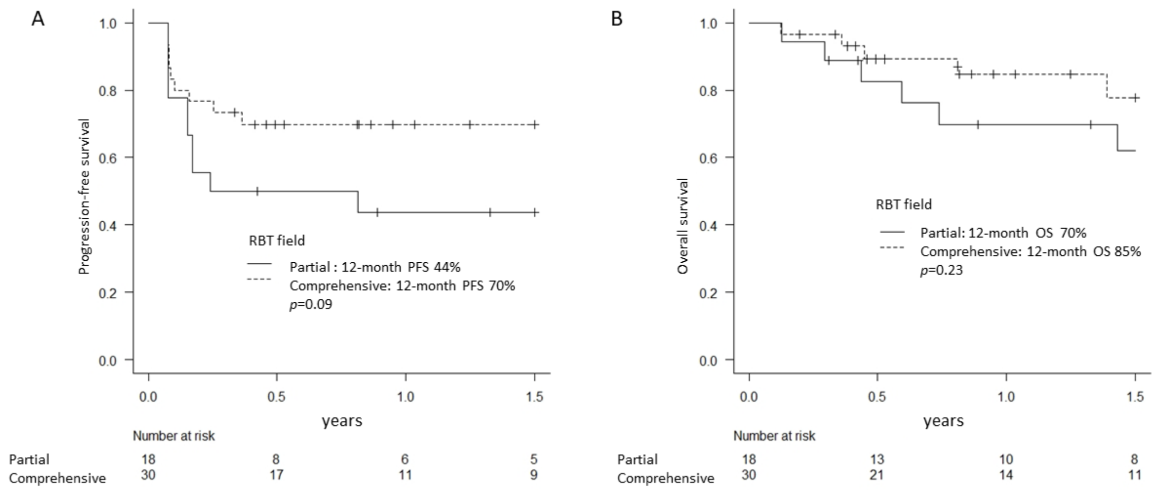

Abbreviations: PFS: progression-free survival; OS: overall survival; and RBT: radiation bridging therapy.

Figure S7: Overall survival according to CAR-T and lymphoma status at infusion

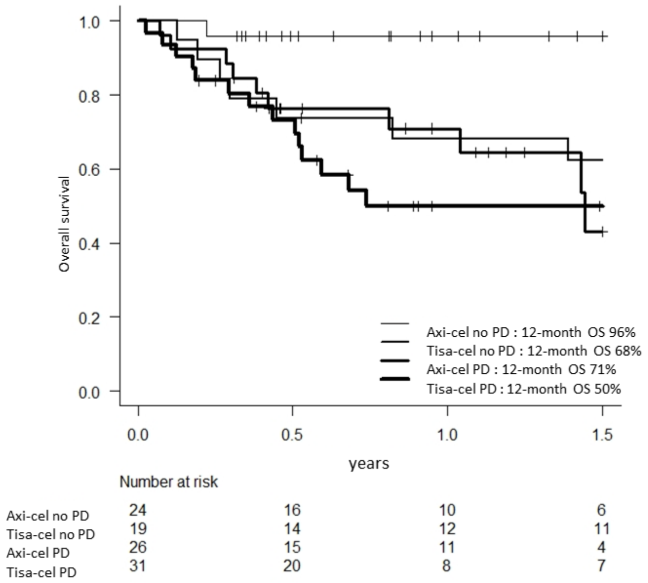

Abbreviations: CAR-T: chimeric antigen receptor T-cell therapy; axi-cel: axicabtagene ciloleucel; tisa-cel: tisagenlecleucel; PD: lymphoma progression prior lymphodepletion; and OS: overall survival. P=0.02 for axi-cel and p=0.07 for tisa-cel.
